# The Role of Exercise Training on Low-Grade Systemic Inflammation in Adults with Overweight and Obesity: A Systematic Review

**DOI:** 10.3390/ijerph182413258

**Published:** 2021-12-16

**Authors:** Paola Gonzalo-Encabo, Gonzalo Maldonado, David Valadés, Carmen Ferragut, Alberto Pérez-López

**Affiliations:** 1 Universidad de Alcalá, Facultad de Medicina y Ciencias de la Salud, Departamento de Ciencias Biomédicas, Área de Educación Física y Deportiva, 28871 Madrid, Spain; gotarossi31@gmail.com (G.M.); david.valades@uah.es (D.V.); carmen.ferragut@uah.es (C.F.); 2 Division of Population Sciences, Department of Medical Oncology, Dana-Farber Cancer Institute, Boston, MA 02215, USA

**Keywords:** exercise immunology, low-grade inflammation, cytokines, fat mass, exercise

## Abstract

Low-grade systemic inflammation leads to critical alterations of several tissues and organs that can promote the appearance of non-communicable diseases, a risk that is increased in adults with obesity. Exercise training may counteract low-grade systemic inflammation, but there is a lack of consensus on how cytokines are modulated by training in adults with obesity. This study aimed of examining the effects of exercise training on circulating pro- and anti-inflammatory cytokines in adults with overweight and obesity, and whether exercise-induced fat mass reduction could mediate that effect. The search was conducted on Medline (Pubmed), SPORTDiscus and Web of Science databases from January 1998 to August 2021, using keywords pertaining to inflammation, exercise, and obesity. A total of 27 studies were selected, in which the circulating concentration levels of cytokines were analyzed. Endurance training (ET) decreased circulating CRP, IL-6 and TNF-α levels. TNF-α was reduced after resistance and concurrent training (CT), while IL-10 increased after resistance training (RT). Changes in IL-10 and CRP coincided with fat mass reduction, while decreased TNF-α levels were concomitant with changes in IL-6 and IL-10. Exercise training may reduce systemic low-grade inflammation profile in adults with overweight and obesity.

## 1. Introduction

Physical inactivity is a serious health problem [1] that causes low-grade systemic inflammation and visceral fat mass accumulation [2]. These two intrinsically linked processes are key factors that influence cardio-metabolic, neurodegenerative, and immune disease development [3]. In people with obesity, low-grade systemic inflammation is related to skeletal muscle mass loss and reduced muscle strength production, metabolic and functional limitations observed in people with multiple comorbidities [4], osteoarthritis [5], or who are hospitalized [6]. Unfortunately, low-grade systemic inflammation still generates more questions than answers and whether exercise training could modulate the chronic inflammatory state of a population with excessive fat mass accumulation or not is one of the critical unsolved questions [7].

Visceral fat mass accumulation stimulates the activation of the innate immunity, which promotes a local response to cellular damage facilitated by increased blood flow, immune cell infiltration (i.e., macrophages) and inflammatory mediator production to repair the damaged tissue, as well as neutralizing any toxic agent produced [8,9]. However, when the inflammatory state persists, adipocytes and immune cells release pro-inflammatory cytokines into the circulation, such as C-reactive protein (CRP), interleukin-6 (IL-6), and tumor necrosis alpha (TNF-α), among others [10]. A chronic presence in the circulation of these pro-inflammatory cytokines is called low-grade systemic inflammation, which contributes to the inflammatory spiral that damage several tissues [11], elevating the risk of non-communicable diseases [12].

In contrast, exercise promotes several anti-inflammatory signals that prevent low-grade systemic inflammation [2,12], by reducing the expression of Toll-like receptors (TLR2 and TLR4) in immune cells [13], attenuating M1 macrophages and CD8^+^ T-cells [14], decreasing macrophages infiltration in adipose tissue and improving adipocytes blood and nutrient supply in visceral fat mass [15]. Some circulatory biomarkers that are released acutely contribute to these anti-inflammatory signals are IL-6 or interleukin-10 (IL-10). Nevertheless, it remains to be elucidated whether exercise training can restrain the inflammatory spiral in patients with overweight or obesity by attenuating low-grade systemic inflammation [7]. Previous narrative reviews have explored the role of physical activity in systemic low-grade inflammation [16,17,18], and some systematic review has examined exercise-induced changes in a particular inflammation marker (e.g., CRP) in general population [19]. However, to our knowledge, no study has analyzed the effect of different doses of exercise on systemic low-grade inflammation. Therefore, this study aimed at examining the effects of exercise training on circulating pro- and anti-inflammatory cytokines in adults with overweight or obesity through a systematic review, as well as to examine whether this effect is mediated by exercise-induced fat mass loss.

## 2. Materials and Methods

### 2.1. Protocol

The present systematic review was developed using guidance from the Preferred Reporting Items for Systematic Reviews and Meta-Analysis protocols (PRISMA-P) [20].

### 2.2. Eligibility Criteria

All types of randomized controlled trials (RCTs) and non-controlled trials examining exercise training intervention (resistance, endurance, high-intensity interval, or concurrent training) on circulating levels of pro- and anti-inflammatory cytokines (CRP, IL-1β, IL-1ra, IL-6, IL-8, IL-10, MCP-1, TNF-α) in adults aged 18 to 65 years old with overweight (>25 kg/m^2^) or obesity (>30 kg/m^2^) were included. Type of exercise, circulating cytokine levels, exercise-induced fat mass loss, and placebo (non-exercise interventions) were used as potential comparators.

### 2.3. Search Strategy, Information Sources and Data Management

Literature was explored using Medline (PubMed), SPORTDiscus and Web of Science, including articles published between January 1998 and August 2021. The search strategy (inflammation AND exercise AND obesity) is presented in Supplementary Table S1, together with an example of the search engine used. A list of reference of included RCTs was elaborated, and an extra scan was performed for additional RCTs. Study authors were contacted when unpublished studies or additional data were required. The identification, screening and abstraction was performed by two authors working independently, while a third author evaluated the quality of the studies, contact with other authors, and explored non-English articles.

### 2.4. Study Selection

After a pilot-testing eligibility criteria for citations, abstract and full-text articles, screening was conducted by two authors working independently, and when any discrepancy regarding eligibility took place, a third author was involved to solve the conflict. The interventions were coded independently, classified into the following broad categories: cytokines, type of exercise, and exercise-induced fat mass loss. Study quality was evaluated independently by two authors; a third authors was not required for this task, since no conflict appears between authors.

Articles were included when they involved physically inactive (<150 min/week), overweight or obese (BMI > 25 kg/m^2^) adult populations (18 to 65 years), who participated in an exercise training program. We examined studies including several biomarkers (CRP, IL-1β, IL-1ra, IL-6, IL-8, IL-10, MCP-1, TNF-α); however, for the purpose of this manuscript, only CRP, IL-6, IL-10 and TNF-α were analyzed and discussed. The remaining biomarker (IL-1β, IL-1ra, IL-8 and MCP-1) are presented in Supplementary Tables S2–S5. While articles were excluded if they involved populations whith overweight or obesity and diagnosed by other cardio-metabolic, immunological, or musculoskeletal pathology, or if the training program lasted less than two weeks or was accompanied by a diet program, articles were also excluded if they included older populations (>65 years) or postmenopausal women.

### 2.5. Data Extraction and Synthesis

The following information was extracted from each study: author, date of publication, sample size, participants’ characteristics, experimental design and procedure, training intervention, and cytokines pre- vs. post-intervention difference. Data were independently extracted by two authors, and any discrepancy was resolved by a third author. Data was collected and presented as the mean ± standard deviation (SD) or mean (SD). When circulating cytokine levels or fat mass loss were provided in figures, values were estimated, and authors were contacted when no data was provided.

Given the heterogeneity of studies (gender, age, duration, intervention, types of training, etc.), it is not possible to combine the studies presented in this systematic review for quantitative analysis (meta-analysis).

### 2.6. Quality Measurement

Study quality assessment was conducted using the PEDro scale [21]. Studies were scored by two authors (Supplementary Table S6). A total score out to 10 was given for each study, 1 was given when the item was satisfied, and 0 when it was not satisfied. When the item was not applicable to that study, NA was used. The first item was excluded from the score calculation. The 27 studies included had a mean PEDro score of 4.8. The most common problems were lack of concealed allocation and a lack of blinding, which is challenging in this type of trial.

## 3. Results

**Figure 1:**
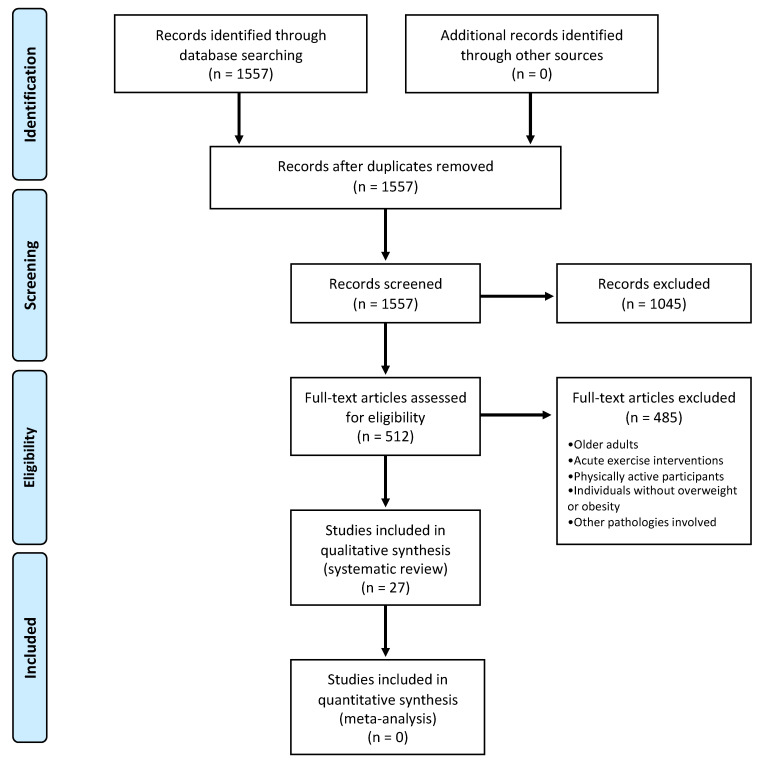
Flow diagram of the systematic review.

Figure 1 illustrates the systematic review flow diagram of the present study. The database search showed 1557 articles, 1045 of which were eliminated after title and abstract examination. The full-text evaluation was performed in 512 articles, and 27 meet the inclusion criteria for systematic review and 0 for meta-analysis. The number of studies included according to cytokines alone and combined with type of exercise are detailed below:

− Protein C-reactive (CRP) (*n* = 13), CRP and endurance training (*n* = 11), CRP and resistance exercise (*n* = 3), CRP and HIIT (*n* = 0) and CRP and concurrent training (*n* = 1).
− Interleukin-1 beta (IL-1β) (*n* = 3), IL-1β and endurance training (*n* = 2), IL-1β and resistance exercise (*n* = 1), IL-1β and HIIT (*n* = 0) and IL-1β and concurrent training (*n* = 0).
− Interleukin-1 receptor alpha (IL-1ra) (*n* = 1), IL-1ra and endurance training (*n* = 1), IL-1ra and resistance training, HITT and concurrent training (*n* = 0).
− Interleukin-6 (IL-6) (*n* = 17), IL-6 and endurance training (*n* = 13), IL-6 and resistance exercise (*n* = 4), IL-6 and HIIT (*n* = 3) and IL-6 and concurrent training (*n* = 3).
− Interleukin-8 (IL-8) (*n* = 2), IL-8 and endurance training (*n* = 1), IL-8 and resistance exercise (*n* = 0), IL-8 and HIIT (*n* = 1) and IL-8 and concurrent training (*n* = 0).
− Interleukin-10 (IL-10) (*n* = 5), IL-10 and endurance training (*n* = 1), IL-10 and resistance exercise (*n* = 2), IL-10 and HIIT (*n* = 2) and IL-10 and concurrent training (*n* = 1).
− Monocyte chemoattractant protein-1 (MCP-1) (*n* = 4), MCP-1 and endurance training (*n* = 3), MCP-1 and resistance exercise (*n* = 0), MCP-1 and HIIT (*n* = 0) and MCP-1 and concurrent training (*n* = 0).
− Tumor necrosis alpha (TNF-α) (*n* = 14), TNF-α and endurance training (*n* = 11), resistance exercise and TNF-α (*n* = 4), TNF-α and HIIT (*n* = 4) and TNF-α and concurrent training (*n* = 1).

### 3.1. Participants and Exercise Interventions’ Characteristics

Circulating CRP levels were analyzed in 13 studies which included 561 participants with overweight or obesity and were involved in a training program [22,23,24,25,26,27,28,29,30,31,32,33,34]. Participants were young and middle-aged males [23,25,27,28,30,32,34] and females [22,25,26,27,28,30,33] who were involved in a randomized control [22,23,26,27,28,31,32,33,34], randomized non-controlled [29,30] or non-randomized non-controlled trial [24,25]. All studies performed an endurance training (ET) program [22,23,24,25,26,27,28,29,30,31,32], except four studies, in which resistance training (RT) [30,33,34] and concurrent training (CT) [30] were analyzed. In ET studies, the training programs consisted of 6 to 12 weeks [23,28,32] or more than 12 weeks [22,24,25,26,27,29,30,31], performed 2–4 [30,32], or 5–7 exercise sessions per week [22,23,24,25,26,27,28,29,31] at moderate intensity [22,23,24,25,27,28,29,30,31,32], except three studies, in which low [26] and vigorous-intensity training were selected [27,31]. In RT and CT studies, the training programs consisted of 8, 22 and 52 weeks [30,33,34], performed 3–4 exercise sessions per week [30,33,34] at moderate intensity [30,34].

Circulating IL-6 lvels were analyzed in 17 studies, in which 439 participants with overweight and obesity were involved in a training program [23,24,25,26,30,33,35,36,37,38,39,40,41,42,43,44,45]. Young and middle-aged men [23,30,35,40,41,42,43,44,45] and women [25,26,30,33,38,39,40,43], older men and women [37] participated in a randomized control [23,26,33,35,37,42], randomized non-controlled [30,36,38,40,43] or a non-randomized non-controlled trial [24,25,39,41,44,45]. Most studies explore the effects of ET on circulating IL-6 levels [23,24,25,26,30,35,36,37,38,39,40,41,43] although RT [30,33,37,44], HIIT [35,40,45] and CT were also evaluated [30,37,42]. In ET studies, the training programs consisted of 2 to 6, 6 to 12 [23,25,35,36,37,39,40,41,43] or more than 12 weeks [24,26,30,38], performed 3–4 [30,35,38,40,43] and 5–7 exercise sessions per week [23,24,25,26,36,37,39,41], at low [26] or moderate intensity [23,24,25,30,35,36,37,38,39,40,41,43]. In RT studies, the training programs consisted of 6 to 12 [37,44] or more than 12 weeks [30,33] performed 2 [33], 3–4 [30,44], or 5 exercise sessions per week [37] at moderate intensity [30,33,37,44]. In the HIIT studies [35,40,45], the duration of the interventions was 2, 6, or 8 weeks, performing 3, 4, and 5 exercise sessions per week in which 4 to 10 series of 1:1 or 2:1 of work:recovery ratio at >75% heart rate of reserve (HRR) or >85% VO_2max_ were carried out. In CT studies, the duration of the programs were 6 to 12 weeks [37] or more than 12 weeks of training [30,42], performed 3 [30,42] or 5 exercise sessions per week [37] at moderate intensity.

Circulating IL-10 levels were analyzed in 5 studies in which 77 participants with overweight or obesity were involved in a training program [23,42,45,46,47]. Participants were young and middle-aged men [23,42,45,46,47] who took part in a randomized control [23,42,46,47] or a non-randomized non-controlled trial [45]. Moreover, different training types were evaluated: ET [23], RT [46,47], HIIT [45,46], and CT [42].

Circulating TNF-α levels were analyzed in 14 studies in which 319 participants with overweight or obesity were involved in a training program [23,24,30,35,36,37,38,39,40,42,44,45,46,48]. Participants were young and middle-aged men [23,24,30,35,37,40,42,44,45,46] and young or middle-aged women [24,30,36,37,38,39,40,48] who were involved in a randomized control [23,35,37,42,46], a randomized non-controlled [30,36,38,40] or a non-randomized non-controlled trial [24,39,44,45]. In ET studies, the training programs consisted of 6 to 12 [23,35,36,37,39,40] or more than 12 weeks of training [24,30,38,48], performed 3–4 [30,35,38,40] or more than 4 exercise sessions per week [23,24,36,37,39,48] at moderate intensity. In RT studies, the training program consisted of between 6–12 [37,44,46] or more than 12 weeks of training [30], performed 3–4 [30,44,46] or 5 exercise sessions per week [37] at moderate [30,37,44] and vigorous intensity [46]. In CT studies, the training program consisted of 12 [37], 22 [30] and 24 [42], performed 3 to 5 days a week at moderate intensity. In HIIT protocols, the duration of the program was less than 6 weeks [45] and between 6–12 weeks [35,40,46], 3 to 5 exercise sessions per week were performed.

### 3.2. C-Reactive Protein (CRP)

Table 1 presents information extracted from the studies that examined circulating CRP concentration levels.

In response to ET, the circulating concentration levels of CRP showed a statistically significant reduction (from −9 to −53%) in 8 of 11 studies (Table 1). When the ET was compared to a control group, four of seven studies reported statistically significant decreases in circulating CRP levels [22,26,27,32], and the reduction of this biomarker was concomitant with a reduction in fat mass in 3 of 4 studies [24,29,30,32]. In response to RT, a statistically significant reduction in circulating CRP levels was found, but no differences were detected when RT was compared to a control group [33], whereas Kolahdouzi et al. (2019) did not find statistically significant changes in CRP levels within or between groups after RT [34].

Furthermore, we examined whether gender could influence these results. A statistically significant reduction in circulating CPR levels was observed in all studies conducted in women, while studies in men showed a statistically significant reduction in CPR levels in two of three (Table 1).

### 3.3. Interleukin-6 (IL-6)

Table 2 presents information extracted from the studies that examined circulating IL-6 concentration levels.

In response to ET, circulating IL-6 showed a statistically significant reduction (from −26 to −32%) in 2 of 13 studies [24,26], but only one study reported statistically significant differences when the ET was compared to a control group [26]. Moreover, in Bruun et al., the reduction in IL-6 levels caused by ET was accompanied by a significant reduction in fat mass [24]. In response to the remaining training types, non-statistically significant differences were found for this biomarker.

Regarding gender-differences, in one of six studies conducted in women was found a statistically significant differences in CPR concentrations [26], while non-significant differences were found in any study performed in men for this biomarker (Table 2).

### 3.4. Interleukin-10 (IL-10)

Table 3 presents information extracted from the studies that examined circulating IL-10 concentration levels.

Different training types were evaluated: HIIT [45,46] ET [23] RT [46,47], and CT [42]. Circulating IL-10 levels reported statistically significant increase in response to HIIT (10%) [46], and RT (6 to 7%) [46,47]. In these three studies, the increase in IL-10 levels was accompanied by a statistically significant decrease in fat mass.

None of the studies performed included a cohort of women participants which satisfied the inclusion criteria, thus, we could not examine the potential influence of gender on the IL-10 response to exercise training.

### 3.5. Tumor Necrosis Alpha (TNF-α)

Table 4 presents information extracted from the studies that examined circulating TNF-α concentration levels.

Circulating concentration levels of TNF-α showed a statistically significant decrease in response to ET (from −21 to −37%) [37,48], RT (−11% and −26%) [37,46], CT [37], and HIIT [46]. When the training intervention was compared to a control group, only ET and CT [37] reported statistically significant differences. While the decrease in TNF-α was only concomitant with a statistically significant reduction in fat mass in two studies [46,48].

Regarding gender-differences, in one of four of the studies conducted in women was found a statistically significant decrease of IL-6 levels [48]. Similarly, in studies on men, one of six studies reported a significant decrease of this biomarker [46].

## 4. Discussion

**Figure 2:**
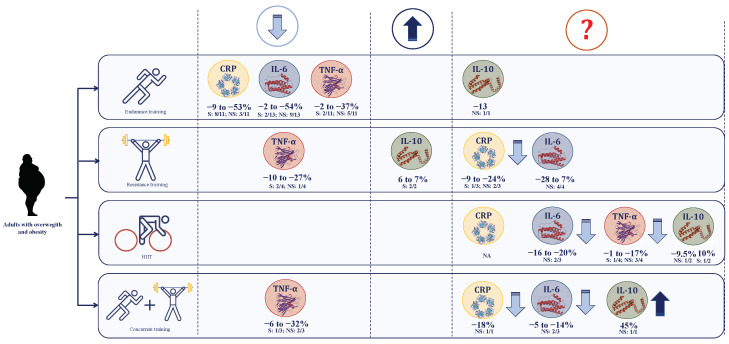
Summary of the results obtained from the systematic review. **Note:** (↑) increased and (↓) decreased circulating concentrations levels. CRP, C-reactive protein; HIIT, High-intensity interval training; IL-6, interleukin-6; IL-10, interleukin-10; NA, not analyzed; NS, studies reporting non-significant differences; S, studies reporting significant differences; TNF-α, tumor necrosis factor alpha.

The present systematic review aimed at evaluating the effects of exercise training on pro- and anti-inflammatory cytokines as low-grade systemic inflammation markers in adults with overweight or obesity. The results are summarized and illustrated in Figure 2. Essentially, circulating concentration levels of CRP, IL-6 and TNF-α are reduced in response to endurance training, while TNF-α is also downregulated after resistance and concurrent training. Moreover, IL-10 increases after resistance training. However, only changes in circulating CRP and IL-10 coincides with a reduction in body fat mass.

CRP (together with IL-6) is considered a convincing marker of low-grade systemic inflammation [49]. Hepatocytes are the primary regulators of CRP, whose synthesis is stimulated by IL-6 production from T-cells [50]. One of the main purposes of CRP is to stimulate phagocytosis activity of macrophages to clear damaged cells or bacteria [50].

Most studies examined the effect of ET on the circulating levels of CRP found a significant decrease from −9 to −53% in 8 of 11 studies (Figure 2 and Table 1). This effect was observed in studies performed ET programs at moderate intensity, but with varied training duration and frequency. Interestingly, the significant reduction of CRP was concomitant with a fat mass diminution [24,29,30,32]. Since no substantial reduction of visceral fat (~5%) is required to promote a reduction of circulating CRP [29], exercise training may indirectly regulate the circulating concentrations levels of this inflammatory marker through stimulating the reduction of body fat mass, consequently promoting a decrease of macrophages infiltration in peripheral tissues.

Initially, IL-6 was categorized as a pro-inflammatory cytokine, given its upregulation during infection. Later, the anti-inflammatory effect of this cytokine was discovered [51]. Although mononuclear cells can be responsible for the circulating fluctuation of IL-6, this does not seem to be the case in response to exercise [52]. In fact, skeletal muscle has been identified as a critical regulator of circulating levels of IL-6 after prolonged exercise training [53].

In the present review, it can be observed that in adults with overweight or obesity, ET training causes a decrease in circulating levels of IL-6 (from −2 to −56%) in 11 of 13 studies, regardless of exercise-induced fat mass loss (Figure 2 and Table 2). These results seem to indicate that depending on the prevailing stimuli, training (anti-inflammatory), or fat mass accumulation (pro-inflammatory), IL-6 bioavailability may be regulated by a different cell type (immune and skeletal muscle cell) facilitating the dual pro- vs. anti-inflammatory function attributed to IL-15 [54], a cytokine of the same family. This dual function of IL-6 may be supported by the consistent but non-significant decrease of IL-6 observed in response not only after endurance, but also after resistance, concurrent, and HIIT training (Figure 2).

In humans, IL-10 synthesis is primarily regulated by monocytes [55], facilitating the inhibition of pro-inflammatory cytokines production in macrophages [56]. Although IL-10 does not seem to be expressed by myocytes [57], in healthy individuals, exercise promotes an upregulation (>80%) of the circulating concentration levels of this cytokine [58]. However, in this review, only RT reported a consistent increase of IL-10, which was concomitant with a reduction in fat mass (Figure 2 and Table 3). It can be argued that in skeletal muscle, the increased expression levels of microRNAs and/or myokines after exercise [59], may increase IL-10 production from monocytes regulating the pro-inflammatory signaling pathway in peripheral tissues, such as adipose tissue, in which macrophages and neutrophils are infiltrated.

Furthermore, in healthy humans, acute bouts of exercise activate myocytes TNF-α expression levels, whereas the small increase of circulating concentrations suggests that skeletal muscle may not be the major regulator of this cytokine [58]. In fact, it seems that monocytes can be the key regulator of this cytokine [60,61], which can be responsible for TNF-α reduction found after ET, RT and HIIT in this review.

Interestingly, a decreased concentration levels of TNF-α has been linked with the downregulation of IL-6 [62] and upregulation of IL-10 [56]. Regarding the TNF-α/IL-6 relationship, most of the analyzed studies reported a coinciding decrease between these two cytokines [24,30,35,37,39]. This decrease of TNF-α and IL-6 can reduce the risk of insulin resistance in a population with obesity [63]. Moreover, a coinciding inverse relationship between TNF-α and IL-10 was also observed in most studies [23,42,46]. Therefore, this evidence may support the regulative role of TNF-α on IL-6 and IL-10 concentration levels in adults with overweight or obesity who experimented an alteration of monocyte metabolism after exercise training. This effect facilitates a diminished inflammatory state by downregulating (IL-6) and upregulating (IL-10).

Despite the regulative role of exercise training on the circulating concentration levels of CRP, IL-6, IL-10 and TNFα observed here, the amount of evidence and the disparity among participants’ characteristics, training protocols, and methods used to determine cytokines concentration levels in blood were major limitations of this systematic review, all of which complicate the understanding of exercise training effects on low-grade systemic inflammation in adults with overweight and obesity. Therefore, further studies with homogeneous characteristics are needed to conduct meta-analysis and to decode the bioavailability of cytokines as markers of the intricate pro- and anti-inflammatory response to training in this population.

## 5. Conclusions

In summary, prolonged doses of endurance, resistance and high-intensity interval training promote a reduction on the circulating concentration of pro-inflammatory cytokines (IL-6, CRP and TNF-α), while resistance training stimulates an increase in anti-inflammatory cytokines (IL-10). This regulative role of exercise training on systemic low-grade inflammation seems to be independent of exercise-induced fat mass loss.

Therefore, the present systematic review may help clinicians to attenuate low-grade systemic inflammation by prescribing the adequate dose of exercise training according to the alteration of pro- and anti-inflammatory cytokines of each patient. However, additional clinical trials are required to further elucidate the regulative role of exercise on the cytokines responsible for low-grade systemic inflammation.

## Figures and Tables

**Table 1:** Effects of training on the circulating concentrations levels of CRP in sedentary adults with overweight or obesity.

Study	Subjects	Experimental Conditions	Training Protocol	Pre- vs. Post-Training Differences	
				Fat Mass (%)	CRP (mg/L)
Esposito et al. 2003	Women (20 to 46 yr) (*n* = 120)	EC1: ET (35.0 ± 2.3 kg/m^2^; *n* = 60) EC2: Control (34.7 ± 2.4 kg/m^2^; *n* = 60)	24 months Aerobic games, swimming and walking	NR	EC1: 3.2 (1.5–8.4) vs. 2.1(0.9–1.9) *^,#^ EC2: 3.4 (1.4–8.3) vs. 3.1 (1.3–8.2)
Bruun et al. 2005	Men (*n* = 11) & Women (*n* = 12) (NR yr)	EC1: ET (45.8 ± 1.9 kg/m^2^; *n* = 23)	15 weeks 5 days/week 2–3 h/session Endurance exercises	EC1: 46.0 ± 2.5 vs. 41.1 ± 2.3 *	EC1: 9.8 ± 1.2 vs. 7.0 ± 1.0 *
Dvorakova-Lorenzova et al. 2005	Women (25 to 35 yr) (*n* = 40)	EC1: ET (31.5 ± 4.0 kg/m^2^; *n* = 40)	9 weeks 6 days/week 45–60 min/session Aerobic exercises 130–135 HR	NR	EC1: 4.31 ± 3.71 vs. 3.01 ± 3.12 *
Marcell et al. 2005	Men (*n* = 20) and Women (*n* = 31) (45.3 ± 8.3 yr)	EC1: ET moderate intensity (33.9 ± 4.9 kg/m^2^; *n* = 17) EC2: ET vigorous intensity (32.5 ± 5.3 kg/m^2^; *n* = 20) EC3: Control (35.3 ± 3.7 kg/m^2^; *n* = 14)	16 weeks 5 days/week 30–40 min/session EC1: walking or jogging 30 min EC2: treadmill 80–90% HRR	EC1: 39.7 ± 8.3 vs. 28.0 ± 2.7 * EC2: 39.8 ± 7.5 vs. 28.0 ± 2.9 * EC3: 43.7 ± 6.4 vs. 43.4 ± 5.9	EC1: 4.9 ± 3.2 vs. 3.9 ± 2.7 EC2: 3.4 ± 3.5 vs. 3.0 ± 3.0 EC3: 5.7 ± 5.1 vs. 4.7 ± 4.6
Jae et al. 2006	Men (*n* = 35) and Women (*n* = 12) (49.6 ± 6.9 yr)	EC1: ET (28.8 ± 2.0 kg/m^2^; *n* = 23) EC2: Control (27.8 ± 2.4 kg/m^2^; *n* = 24)	3 months >5 days/week 50–60 min/session Aerobic exercises 60–80% HR_max_	NR	EC1: 0.16 ± 0.13 vs. 0.09 ± 0.07 * EC2: 0.10 ± 0.08 vs. 0.15 ± 0.17
Olson et al. 2007	Women (24 to 44 yr) (*n* = 28)	EC1: RT (26.9 ± 3.0 kg/m^2^; *n* = 16) EC2: Control (27 ± 3 kg/m^2^; *n* = 12)	1 year 2 days/week ~9 exercises (3 × 8−10)	EC1: 43.4 ± 3.7 vs. 41.5 ± 4.7 EC2: 44.8 ± 4.4 vs. 43.0 ± 4.9	EC1: 3.3 ± 0.4 vs. 3.0 ± 0.4 * EC2: 3.2 ± 0.4 vs. 3.4 ± 0.4
Arikawa et al. 2010	Women (*n* = 319) (18 to 30 yr)	EC1: ET (NR; *n* = 166) EC2: Control (NR; *n* = 153)	16 weeks 5 days/week 45 min/session Endurance training 65–85% HR_max_	EC1: 36.4 ± 8.7 vs. NR EC2: 36.1 ± 8.3 vs. NR	EC1: 5.02 (4.17–6.03) vs. 4.32 (3.60–5.19) *^,#^ EC2: 3.94 (3.25–4.76) vs. 3.90 (3.22–4.73)
Moghadasi et al. 2012	Men (*n* = 16) (Middle-aged)	EC1: ET (30.9 ± 2.1 kg/m^2^; *n* = 8) EC2: Control (32.0 ± 5.3 kg/m^2^; *n* = 8)	12 weeks 4 days/week 45 min/session Treadmill 75–80% VO_2max_	EC1: 29.5 ± 3.1 vs. 27.2 ± 3.7 *^,#^ EC2: 31.4 ± 5.5 vs. 31.4 ± 5.5	EC1: ~2.25 ± 0.75 vs. ~0.85 ± 0.65 *^,#^ EC2: ~2.8 ± 0.8 vs. ~3.1 ± 0.8
Auerbach et al. 2013	Men (20 to 40 yr) (*n* = 48)	EC1: ET (28.1 ± 1.3 kg/m^2^; *n* = 12) EC2: Control (28.1 ± 1.3 kg/m^2^; *n* = 12)	12 weeks 7 days/week 65–85% HRR (600 kcal)	EC1: 31.3 ± 4.1 vs. 29.4 ± 3.8 * EC2: 31.3 ± 4.1 vs. 31.1 ± 3.5	EC1: 1.4 ± 0.5 vs. 1.6 ± 0.7 EC2: 1.4 ± 0.5 vs. 1.4 ± 0.6
Loria-Kohen et al. 2013	Men (*n* = 46) and Women (*n* = 46) (18 to 50 yr)	EC1: RT (29.5 ± 2.0 kg/m^2^; *n* = 19) EC2: ET (28.9 ± 1.7 kg/m^2^; *n* = 25) EC3: Concurrent training (28.3 ± 1.5 kg/m^2^; *n* = 22)	22 weeks 3 days/week EC1: 8 exercise (1 ×15 at 50–60% 15 RM (R = 15 s) EC2: treadmill or cycle 50–60% HRR EC3: treadmill or cycle 50–60% HRR + 8 exercise (1 × 15 at 50–60% 15 RM (R = 15 s)	EC1: 40.2 ± 6.7 vs. 36.1 ± 7.7 * EC2: 39.8 ± 5.6 vs. 35.3 ± 6.8 * EC3: 37.5 ± 6.0 vs. 30.0 ± 7.6 *	EC1: 1.89 (0.69–3.62) vs. 1.45 (0.79–3.17) EC2: 2.09 (1.00–5.07) vs. 1.02 (0.79–4.05) * EC3: 0.96 (0.79–2.08) vs. 0.79 (0.79–2.01)
Khoo et al. 2015	Men (~42.6 yr) (*n* = 80)	EC1: ET (32.1 ± 2.6 kg/m^2^; *n* = 40)	24 weeks 3–7 days/week 45–60 min/session Aerobic exercises 60–80% HRR	EC1: 34.7 ± 5.5 vs. 31.0 ± 3.4 *	EC1: 3.94 ± 3.56 vs. 1.83 ± 3.13 *
Gram et al. 2017	Men (*n* = 46) and Women (*n* = 44) (20 to 45 yr)	EC1: ET moderate intensity (29.2 (28.5–29.9) kg/m^2^; *n* = 31) EC2: ET vigorous intensity (30.1 (29.2–30.5) kg/m^2^; *n* = 24) EC3: Control (30.2 (28.9–31.5) kg/m^2^; *n* = 16)	6 months EC1: 50% VO_2peak_ EC2: 70% VO_2peak_ EC3: Bike	NR	EC1: ~1.5 ± 1.3 vs. ~0.7 ± 0.5 *^,#^ EC2: ~1.1 ± 0.4 vs. ~0.8 ± 0.4 EC3: ~1.4 ± 1.9 vs. ~1.6 ± 2.1
Kolahdouzi et al. 2019	Men (*n* = 30)	EC1: Control (31.1 ± 3.2 kg/m^2^; *n* = 15) EC2: RT (30.12 ± 2.99 kg/m^2^; *n* = 15)	8 weeks 3–7 days/week 60 min/session Progressive resistance training circuit: 8 exercises (2−4 × 8−12 at 65–85% RM (R = 15 s between exercises and 3 min between series)	NR	EC1: ~1.5 ± 0.8 vs. ~1.5 ± 0.8 EC2: ~2.1 ± 1.3 vs. ~1.6 ± 1.2

EC = experimental condition; ET = Endurance training; HRR = heart rate reserve; HR_max_ = maximal heart rate; NR = non-reported; R = rest between series; RM = maximal repetition; RT = Resistance training; VO_2max_ = maximal oxygen consumption; ~ = estimated data; some data are presented as median (interquartile range); * *p* < 0.05 within group comparison; ^#^ *p* < 0.05 between groups comparison (vs control). Data are shown as mean ± SD.

**Table 2:** Effects of training on the circulating concentrations of IL-6 in sedentary adults with overweight or obesity.

Study	Subjects	Experimental Conditions	Training Protocol	Pre- vs. Post-Training Differences	
				Fat Mass (%)	IL-6 (pg/mL)
Esposito et al. 2003	Women (20 to 46 yr) (*n* = 120)	EC1: ET (35.0 ± 2.3 kg/m^2^; *n* = 60) EC2: Control (34.7 ± 2.4 kg/m^2^; *n* = 60)	24 months Aerobic games, swimming, and walking	NR	EC1: 4.3 (1.9–9.0) vs. 2.9 (1.1–6.5) *^,#^ EC2: 4.1 (2.0–9.0) vs. 3.8 (2.1–8.9)
Bruun et al. 2005	Men (*n* = 11) & Women (*n* = 12) (NR yr)	EC1: ET (45.8 ± 1.9 kg/m^2^; *n* = 23)	15 weeks 5 days/week 2–3 h/session Moderate intensity (NR)	EC1: 46.0 ± 2.5 vs. 41.1 ± 2.3 *	EC1: 4.6 ± 0.6 vs. 3.4 ± 0.6 *
Dvorakova-Lorenzova et al. 2005	Women (25 to 35 yr) (*n* = 40)	EC1: ET (31.5 ± 4.0 kg/m^2^; *n* = 40)	9 weeks 6 days/week 45–60 min/session Aerobic exercises 130–135 HR	NR	EC1: 9.01 ± 6.47 vs. 11.25 ± 7.21
Klimcakova et al. 2006	Men (50.4 ± 2.3 yr) (*n* = 12)	EC1: RT (33.6 ± 1.2 kg/m^2^; *n* = 12)	12 weeks 3 days/week 60 min/session 17 exercises, 1 × 12−15 at 60–70% RM	EC1: 31.6 ± 4.9 vs. 30.1 ± 4.2	EC1: 1.4 ± 0.7 vs. 1.5 ± 0.6
Polak et al. 2006	Women (40.4 ± 6.7 yr) (*n* = 25)	EC1: ET (32.2 ± 2.2 kg/m^2^; *n* = 25)	12 weeks 5 days/week 45 min/session Cycling at 55–65% VO_2max_	EC1: 38.8 ± 4.2 vs. 36.3 ± 4.6 *	EC1: 3.1 ± 3.7 vs. 1.4 ± 1.5
Olson et al. 2007	Women (24 to 44 yr) (*n* = 28)	EC1: RT (26.9 ± 3.0 kg/m^2^; *n* = 16) EC2: Control (27 ± 3 kg/m^2^; *n* = 12)	1 year 2 days/week ~9 exercises (3 × 8−10)	EC1: 43.4 ± 3.7 vs. 41.5 ± 4.7 EC2: 44.8 ± 4.4 vs. 43.0 ± 4.9	EC1: 2.8 ± 1.8 vs. 2.0 ± 0.7 EC2: 2.4 ± 0.9 vs. 2.5 ± 1.3
Christiansen et al. 2010	Men (*n* = 40) and Women (*n* = 34) (18 to 45 yr)	EC1: ET (33.3 ± 4.0 kg/m^2^; *n* = 25)	12 weeks 3 days/week 60–75 min/session (500–600 kcal)	NR	EC1: 3.2 ± 2.4 vs. 2.5 ± 1.6
Leggate et al. 2012	Men (23.7 ± 5.2 yr) (*n* = 12)	EC1: HIIT (29.1 ± 3.1 kg/m^2^; *n* = 12)	2 weeks 3 days/week HIIT 10 × (4 min 85% VO_2peak_ + NR R)	NR	EC1: 3.1 ± 3.0 vs. 2.6 ± 2.2
Auerbach et al. 2013	Men (20 to 40 yr) (*n* = 48)	EC1: ET (28.1 ± 1.3 kg/m^2^; *n* = 12) EC2: Control (28.1 ± 1.3 kg/m^2^; *n* = 12)	12 weeks 7 days/week 65–85% HRR (600 kcal)	EC1: 31.3 ± 4.1 vs. 29.4 ± 3.8 * EC2: 31.3 ± 4.1 vs. 31.1 ± 3.5	EC1: 15 ± 27 vs. 14.6 ± 25.2 EC2: 15 ± 27 vs. 12.7 ± 26
Besse-Patin et al. 2013	Men (35.4 ± 1.5 yr) (*n* = 11)	EC1: ET (32.6 ± 2.3 kg/m^2^; *n* = 11)	8 weeks 5 days/week 45–60 min/session Cycling or running 35–85% VO_2max_	NR	EC1: ~6.2 ± 4.0 vs. ~5.0 ± 2.0
Ho et al. 2013	Men (*n* = 10) & Women (*n* = 87) (40 to 65 yr)	EC1: ET (32.7 (25.0–45.6) kg/m^2^; *n* = 15) EC2: RT (33.0 (25.8–44.6) kg/m^2^; *n* = 16) EC3: Concurrent training (33.3 (23.4–40.2) kg/m^2^; *n* = 17) EC4: Control (32.4 (26.0–48.0) kg/m^2^; *n* = 16)	12 weeks 5 days/week 30 min/session EC1: treadmill at 60% HRR EC2: 5 exercises (4 × 8−12 at 10 RM, R = 60 s) EC3: 15 min treadmill 60% HRR + 5 exercises (2 × 8−12 at 10 RM, R = 60 s)	EC1: 44.6 (30.7–52.5) vs. NR EC2: 43.7 (34.6–52.2) vs. NR EC3: 45.8 (28.8–55.5) vs. NR EC4: 46.5 (35.9–9.9) vs. NR	EC1: 2.5 (0.0–8.5) vs. ~2.0 ± 0.7 EC2: 2.3 (0.0–7.4) vs. ~1.7 ± 0.5 EC3: 2.3 (0.0–12.4) vs. ~2.8 ± 1.1 EC4: 3.0 (0.0–13.1) vs. ~2.3 ± 0.9
Lakhdar et al. 2013	Women (~38 yr) (*n* = 30)	EC1: ET (33.5 ± 3.7 kg/m^2^; *n* = 10)	24 weeks 3 days/week Treadmill 30–45 min at 55–80% HR_max_	EC1: 41.3 ± 1.2 vs. 41.1 ± 1.3	EC1: 6.87 ± 0.24 vs. 6.75 ± 0.27
Loria-Kohen et al. 2013	Men (*n* = 46) and Women (*n* = 46) (18 to 50 yr)	EC1: RT (29.5 ± 2.0 kg/m^2^; *n* = 19) EC2: ET (28.9 ± 1.7 kg/m^2^; *n* = 25) EC3: Concurrent training (28.3 ± 1.5 kg/m^2^; *n* = 22)	22 weeks 3 days/week EC1: 8 exercise (1 × 15 at 50–60% 15 RM (R = 15 s) EC2: treadmill or cycle 50–60% HRR EC3: treadmill or cycle 50–60% HRR + 8 exercise (1 × 15 at 50–60% 15 RM (R = 15 s)	EC1: 40.2 ± 6.7 vs. 36.1 ± 7.7 * EC2: 39.8 ± 5.6 vs. 35.3 ± 6.8 * EC3: 37.5 ± 6.0 vs. 30.0 ± 7.6 *	EC1: 2.60 (2.28–3.75) vs. 2.70 (1.97–4.90) EC2: 4.89 (3.42–7.89) vs. 4.44 (3.32–5.34) EC3: 2.45 (0.13–3.65) vs. 2.11 (0.11–3.67)
Ahmadizad et al. 2015	Men (25 ± 1 yr) (*n* = 30)	EC1: HIIT (27.6 ± 1.9 kg/m^2^; *n* = 10) EC2: ET (27.6 ± 1.9 kg/m^2^; *n* = 10) EC3: Control (27.6 ± 1.9 kg/m^2^; *n* = 10)	6 weeks 3 days/week EC1: 8 × (4–6 min at 90% VO_2max_ + 2–3 min R) EC2: 30–70 min at 50–60% VO_2max_	EC1: 24.2 ± 2.1 vs. 23.2 ± 2.1 *^,#^ EC2: 26.2 ± 2.3 vs. 25.1 ± 2.3 *^,#^ EC3: 25.4 ± 2.1 vs. 26.4 ± 2.0	EC1: ~0.5 ± 0.3 vs. ~0.4 ± 0.3 EC2: ~0.5 ± 0.4 vs. ~0.4 ± 0.3 EC3: ~0.5 ± 0.4 vs. ~0.5 ± 0.4
Brunelli et al. 2015	Men (48.7 ± 1.0 yr) (*n* = 30)	EC1: Concurrent training (31.0 ± 0.4 kg/m^2^; *n* = 17) EC2: Control (31.0 ± 0.4 kg/m^2^; *n* = 13)	24 weeks 3 days/week 6 exercise (3 × 6−10 rep RM (R = 1 min)) + 30 min running 50–85% VO_2peak_	EC1: 36.0 ± 1.4 vs. 28.6 ± 1.6 * EC2: 32.3 ± 1.7 vs. 31.1 ± 1.8	EC1: ~1.9 ± 0.2 vs. ~1.8 ± 0.1 EC2: ~1.6 ± 0.1 vs. ~2.4 ± 0.2
Vella et al. 2017	Men (*n* = 7) and Women (*n* = 10) (18 to 44 yr)	EC1: HIIT (29.9 ± 3.3 kg/m^2^; *n* = 8) EC2: ET (33.1 ± 6.0 kg/m^2^; *n* = 9)	8 weeks 4 days/week 30 min/session EC1: 10 × (60 s at 75–80% HRR + 60 s at 35–40% HRR) EC2: 55–59% HRR	EC1: 35.2 ± 6.8 vs. NR EC2: 35.3 ± 7.2 vs. NR	EC1: 0.5 ± 0.1 vs. 1.0 ± 0.2 ^#^ EC2: 1.0 ± 0.3 vs. 0.4 ± 0.2
Duzova et al. 2018	Women (~38.5 yr) (*n* = 25)	EC1: ET-steps (25.7 ± 0.8 kg/m^2^; *n* = 10) EC2: ET-walking (29.0 ± 1.3 kg/m^2^; *n* = 15)	12 weeks 5 days/week EC1: steps aerobics 40–60 min 80% HR_max_ EC2: jogging-walking 50–60 min 80% HR_max_	EC1: 31.23 ± 1.76 vs. 27.33 ± 1.96 EC2: 35.81 ± 1.76 vs. 32.67 ± 1.91	EC1: 11.24 ± 1.3 vs. 10.5 ± 1.4 EC2: 9.40 ± 0.67 vs. 16.6 ± 6.1

EC = experimental condition; ET = Endurance training; HIIT = high intensity interval training; HRR = heart rate reserve; HR_max_ = maximal heart rate; R = rest between series; RM = maximal repetition; RT = Resistance training; VO_2peak_ = peak oxygen uptake; NR = not reported; ~ = estimated data. * *p* < 0.05 within group comparison; ^#^ *p* < 0.05 between groups comparison (vs control); some data are presented as median (interquartile range). Data are shown as mean ± SD.

**Table 3:** Effects of training on the circulating concentrations of IL-10 in sedentary adults with overweight or obesity.

Study	Subjects	Experimental Conditions	Training Protocol	Pre- vs. Post-Training Differences	
				Fat Mass (%)	IL-10 (pg/mL)
Leggate et al. 2012	Men (23.7 ± 5.2 yr) (*n* = 12)	EC1: HIIT (29.1 ± 3.1 kg/m^2^; *n* = 12)	2 weeks 3 days/week HIIT 10 × (4 min 85% VO_2peak_ + NR R)	NR	EC1: 2.1 ± 0.6 vs. 1.9 ± 0.6
Auerbach et al. 2013	Men (20 to 40 yr) (*n* = 48)	EC1: ET (28.1 ± 1.3 kg/m^2^; *n* = 12) EC2: Control (28.1 ± 1.3 kg/m^2^; *n* = 12)	12 weeks 7 days/week 65–85% HRR (600 kcal)	EC1: 31.3 ± 4.1 vs. 29.4 ± 3.8 * EC2: 31.3 ± 4.1 vs. 31.1 ± 3.5	EC1: 11 ± 11 vs. 9.5 ± 8.5 EC2: 11 ± 11 vs. 15.5 ± 16.2
Nikseresht et al. 2014	Men (34 to 46 yr) (*n* = 32)	EC1: HIIT (NR; *n* = 12) EC2: RT (NR; *n* = 12) EC3: Control (NR; *n* = 10)	12 weeks 3 days/week 45–60 min/session EC1: 4 × (4 min 80–90% HR_max_ + 3 min 55–65% HR_max_) EC2: 1–4 s/2–20 rep 40–95% RM (R = 1–7 min)	EC1: ~30.4 vs. ~27.9 *^,#^ EC2: ~30.0 vs. ~27.4 *^,#^ EC3: ~29.5 vs. ~30.1	EC1: 6.68 ± 0.82 vs. 7.32 ± 0.99 * EC2: 7.06 ± 0.71 vs. 7.46 ± 0.64 * EC3: 7.31 ± 1.06 vs. 7.17 ± 0.81
Brunelli et al. 2015	Men (48.7 ± 1.0 yr) (*n* = 30)	EC1: Concurrent training (31.0 ± 0.4 kg/m^2^; *n* = 17) EC2: Control (31.0 ± 0.4 kg/m^2^; *n* = 13)	24 weeks 3 days/week 6 exercise (3 × 6−10 rep RM (R = 1 min)) + 30 min running 50–85% VO_2peak_	EC1: 36.0 ± 1.4 vs. 28.6 ± 1.6 * EC2: 32.3 ± 1.7 vs. 31.1 ± 1.8	EC1: ~0.22 ± 0.01 vs. ~0.32 ± 0.02 EC2: ~0.38 ± 0.04 vs. ~0.22 ± 0.02
Nikseresht et al. 2018	Men (~39.5 yr) (*n* = 22)	EC1: RT (*n* = 12) EC2: Control (*n* = 10)	12 weeks 3 days/week 10 exercise (1 × 20 at 40 to 90%1 RM)	EC1: 30.7 ± 1.8 vs. 28.4 ± 1.9 *^,#^ EC2: 29.7 ± 1.2 vs. 30.1 ± 1.7	EC1: 7.06 ± 0.71 vs. 7.58 ± 0.67 * EC2: 7.46 ± 0.64 vs. 7.17 ± 0.81

EC = experimental condition; ET = Endurance training; HIIT = high intensity interval training; HRR = heart rate reserve; HR_max_ = maximal heart rate; NR = non-reported; R = rest between series; RM = maximal repetition; VO_2peak_ = peak oxygen uptake; ~ = estimated data; * *p* < 0.05 within group comparison; ^#^ *p* < 0.05 between group comparison. Data are shown as mean ± SD.

**Table 4:** Effects of training on the circulating concentrations of TNF-α in sedentary adults who were overweight or obese.

Study	Subjects	Experimental Conditions	Training Protocol	Pre- vs. Post-Training Differences	
				Fat Mass (%)	TNF-α (pg/mL)
Bruun et al. 2005	Men (*n* = 11) & Women (*n* = 12) (NR yr)	EC1: ET (45.8 ± 1.9 kg/m^2^; *n* = 23)	15 weeks 5 days/week 2–3 h/session Moderate intensity (NR)	EC1: 46.0 ± 2.5 vs. 41.1 ± 2.3 *	EC1: 1.0 ± 0.1 vs. 1.0 ± 0.2
Klimcakova et al. 2006	Men (50.4 ± 2.3 yr) (*n* = 12)	EC1: RT (33.6 ± 1.2 kg/m^2^; *n* = 12)	12 weeks 3 days/week 60 min/session 17 exercises, 1 × 12−15 at 60–70% RM	EC1: 31.6 ± 4.9 vs. 30.1 ± 4.2	EC1: 2.0 ± 1.5 vs. 2.3 ± 2.2
Kondo et al. 2006	Women (18 to 23 yr) (*n* = 16)	EC1: ET (29.5 ± 2.7 kg/m^2^; *n* = 8) EC2: Control (21.9 ± 3.2 kg/m^2^; *n* = 8)	28 weeks 4–5 days/week >30 min/session (300–400 kcal) 60–70% HRR	EC1: 29.8 ± 0.9 vs. 25.6 ± 4.6 * EC2: 22.5 ± 8.9 vs. 18.5 ± 3.2 *	EC1: 7.6 ± 2.3 vs. 4.8 ± 1.2 * EC2: 2.3 ± 0.9 vs. 2.1 ± 1.4
Polak et al. 2006	Women (40.4 ± 6.7 yr) (*n* = 25)	EC1: ET (32.2 ± 2.2 kg/m^2^; *n* = 25)	12 weeks 5 days/week 45 min/session Cycling at 55–65% VO_2max_	EC1: 38.8 ± 4.2 vs. 36.3 ± 4.6 *	EC1: 6.1 ± 7.6 vs. 4.8 ± 4.5
Leggate et al. 2012	Men (23.7 ± 5.2 yr) (*n* = 12)	EC1: HIIT (29.1 ± 3.1 kg/m^2^; *n* = 12)	2 weeks 3 days/week HIIT 10 × (4 min 85% VO_2peak_ + NR R)	NR	EC1: 1.3 ± 0.4 vs. 1.3 ± 0.5
Auerbach et al. 2013	Men (20 to 40 yr) (*n* = 48)	EC1: ET (28.1 ± 1.3 kg/m^2^; *n* = 12) EC2: Control (28.1 ± 1.3 kg/m^2^; *n* = 12)	12 weeks 7 days/week 65–85% HRR (600 kcal)	EC1: 31.3 ± 4.1 vs. 29.4 ± 3.8 * EC2: 31.3 ± 4.1 vs. 31.1 ± 3.5	EC1: 7.1 ± 2.4 vs. 8.0 ± 3.1 EC2: 7.1 ± 2.4 vs. 6.7 ± 2.0
Ho et al. 2013	Men (*n* = 10) & Women (*n* = 87) (40 to 65 yr)	EC1: ET (32.7 (25.0–45.6) kg/m^2^; *n* = 15) EC2: RT(33.0 (25.8–44.6) kg/m^2^; *n* = 16) EC3: Concurrent training (33.3 (23.4–40.2) kg/m^2^; *n* = 17) EC4: Control (32.4 (26.0–48.0) kg/m^2^; *n* = 16)	12 weeks 5 days/week 30 min/session EC1: treadmill at 60% HRR EC2: 5 exercises (4 × 8–12 at 10 RM, R = 60 s) EC3: 15 min treadmill 60% HRR + 5 exercises (2 × 8–12 at 10 RM, R = 60 s)	EC1: 44.6 (30.7–52.5) vs. NR EC2: 43.7 (34.6–52.2) vs. NR EC3: 45.8 (28.8–55.5) vs. NR EC4: 46.5 (35.9–9.9) vs. NR	EC1: 14.6 (8.1–23.3) vs. ~11.6 ± 1.0 * EC2: 12.0 (6.4–20.0) vs. ~8.8 ± 0.5 * EC3: 12.6 (4.3–25.8) vs. ~8.6 ± 1.0 *^,#^ EC4: 10.2 (4.9–17.0) vs. ~9.6 ± 0.5
Lakhdar et al. 2013	Women (~38 yr) + (*n* = 30)	EC1: ET (33.5 ± 3.7 kg/m^2^; *n* = 10)	24 weeks 3 days/week Treadmill 30–45 min at 55–80% HR_max_	EC1: 41.3 ± 1.2 vs. 41.1 ± 1.3	EC1: 2.62 ± 0.29 vs. 2.45 ± 0.26
Loria-Kohen et al. 2013	Men (*n* = 46) and Women (*n* = 46) (18 to 50 yr)	EC1: RT (29.5 ± 2.0 kg/m^2^; *n* = 19) EC2: ET (28.9 ± 1.7 kg/m^2^; *n* = 25) EC3: Concurrent training (28.3 ± 1.5 kg/m^2^; *n* = 22)	22 weeks 3 days/week EC1: 8 exercises (1 × 15 at 50–60% 15 RM (R = 15 s) EC2: treadmill or cycle 50–60% HRR EC3: treadmill or cycle 50–60% HRR + 8 exercise (1 × 15 at 50–60% 15 RM (R = 15 s)	EC1: 40.2 ± 6.7 vs. 36.1 ± 7.7 * EC2: 39.8 ± 5.6 vs. 35.3 ± 6.8 * EC3: 37.5 ± 6.0 vs. 30.0 ± 7.6 *	EC1: 4.96 (4.18–5.48) vs. 4.44 (3.98–5.21) EC2: 3.60 (3.14–4.87) vs. 3.31 (2.66–4.38) EC3: 4.68 (0.91–7.33) vs. 4.41 (0.73–6.17)
Nikseresht et al. 2014	Men (34 to 46 yr) (*n* = 32)	EC1: HIIT (NR; *n* = 12) EC2: RT (NR; *n* = 12) EC3: Control (NR; *n* = 10)	12 weeks 3 days/week 45–60 min/session EC1: 4 × (4 min 80–90% HR_max_ + 3 min 55–65% HR_max_) EC2: 1–4 s/2–20 rep 40–95% RM (R = 1–7 min)	EC1: ~30.4 vs. ~27.9 *^,#^ EC2: ~30.0 vs. ~27.4 *^,#^ EC3: ~29.5 vs. ~30.1	EC1: 2.99 ± 0.64 vs. 2.60 ± 0.54 * EC2: 3.00 ± 0.46 vs. 2.66 ± 0.53 * EC3: 2.90 ± 0.74 vs. 2.96 ± 0.64
Ahmadizad et al. 2015	Men (25 ± 1 yr) (*n* = 30)	EC1: HIIT (27.6 ± 1.9 kg/m^2^; *n* = 10) EC2: ET (27.6 ± 1.9 kg/m^2^; *n* = 10) EC3: Control (27.6 ± 1.9 kg/m^2^; *n* = 10)	6 weeks 3 days/week EC1: 8 × (4–6 min at 90% VO_2max_ + 2–3 min R) EC2: 30–70 min at 50–60% VO_2max_	EC1: 24.2 ± 2.1 vs. 23.2 ± 2.1 *^,#^ EC2: 26.2 ± 2.3 vs. 25.1 ± 2.3 *^,#^ EC3: 25.4 ± 2.1 vs. 26.4 ± 2.0	EC1: ~3.0 ± 0.8 vs. ~2.5 ± 1.0 EC2: ~2.8 ± 1.5 vs. ~2.7 ± 1.6 EC3: ~2.8 ± 1.3 vs. ~2.9 ± 1.3
Brunelli et al. 2015	Men (48.7 ± 1.0 yr) (*n* = 30)	EC1: Concurrent training (31.0 ± 0.4 kg/m^2^; *n* = 17) EC2: Control (31.0 ± 0.4 kg/m^2^; *n* = 13)	24 weeks 3 days/week 6 exercise (3 × 6−10 rep RM (R = 1 min)) + 30 min running 50–85% VO_2peak_	EC1: 36.0 ± 1.4 vs. 28.6 ± 1.6 * EC2: 32.3 ± 1.7 vs. 31.1 ± 1.8	EC1: ~2.4 ± 0.1 vs. ~1.9 ± 0.1 EC2: ~2.3 ± 0.1 vs. ~3.5 ± 0.3 *
Vella et al. 2017	Men (*n* = 7) and Women (*n* = 10) (18 to 44 yr)	EC1: HIIT (29.9 ± 3.3 kg/m^2^; *n* = 8) EC2: ET (33.1 ± 6.0 kg/m^2^; *n* = 9)	8 weeks 4 days/week 30 min/session EC1: 10 × (60 s at 75–80% HRR + 60 s at 35–40% HRR) EC2: 55–59% HRR	EC1: 35.2 ± 6.8 vs. NR EC2: 35.3 ± 7.2 vs. NR	EC1: 2.1 ± 0.2 vs. 2.1 ± 0.1 EC2: 2.0 ± 0.1 vs. 2.1 ± 0.1
Duzova et al. 2018	Women (~38.5 yr) (*n* = 25)	EC1: ET-steps (25.7 ± 0.8 kg/m^2^; *n* = 10) EC2: ET-walking (29.0 ± 1.3 kg/m^2^; *n* = 15)	12 weeks 5 days/week EC1: steps aerobics 40–60 min 80% HR_max_ EC2: jogging-walking 50–60 min 80% HR_max_	EC1: 31.23 ± 1.76 vs. 27.33 ± 1.96 EC2: 35.81 ± 1.76 vs. 32.67 ± 1.91	EC1: 95.6 ± 18.6 vs. 102.9 ± 18.5 *^,#^ EC2: 57.5 ± 14.3 vs. 56.1 ± 13.8

EC = experimental condition; ET = Endurance training; HIIT = high intensity interval training; HRR = heart rate reserve; HR_max_ = maximal heart rate; NR = not reported; R = rest between series; RM = maximal repetition; RT = Resistance training; VO_2peak_ = peak oxygen uptake; ~ = estimated data; * *p* < 0.05 within group comparison; ^#^ *p* < 0.05 between group comparison. Data are shown as mean ± SD.

## Supplementary Materials

The following are available online at https://www.mdpi.com/article/10.3390/ijerph182413258/s1, Supplementary Table S1: Keywords used in database search strategy; Supplementary Table S2: Effects of training on the circulating concentrations of IL-1β in sedentary adults with overweight or obesity; Supplementary Table S3: Effects of training on the circulating concentrations of IL-1ra in sedentary adults with overweight and obesity; Supplementary Table S4: Effects of training on the circulating concentrations of IL-1ra in sedentary adults with overweight and obesity; Supplementary Table S5: Effects of training on the circulating concentrations of MCP-1 in sedentary adults with overweight and obesity; Supplementary Table S6: PEDro Scale for quality assessment of the studies included in the systematic review.
