# Supplementary material for: The Role of Exercise Training on Low-Grade Systemic Inflammation in Adults with Overweight and Obesity: A Systematic Review
Source: Int J Environ Res Public Health. 2021 Dec 16;18(24):13258. doi: 10.3390/ijerph182413258 (PMC8703787; doi:10.3390/ijerph182413258)
Supplement: Supplementary file 1 [file ijerph-18-13258-s001.zip › Supplementary Table S1-S6-MDPI.pdf]

Supplementary Table S1. Keywords used in database search strategy

Concept 1: Inflammation	Concept 2: Exercise	Concept 3: Obesity
CRP	Aerobic exercise	Fat accumulation
IL-6	Aerobic training	Obesity
IL-10	Endurance exercise	Overweight
Inflammation	Endurance training	
TNF-α	Exercise	
IL-1ra	Resistance exercise	
IL-1 β	Resistance training	
IL-8	Training	
MCP-1		

IL-1β, interleukin-1; IL-6, interleukin-6; IL-8, interleukin-8; IL-10, interleukin-10; MCP-1, monocyte chemoattractant protein 1; PCR, protein C-reactive; TNF-α, tumor necrosis factor alpha.

Example of a search strategy for protein C-reactive (CRP):

(((“CRP”[Mesh] OR “CRP”[Title/Abstract]) OR (“protein C-reactive”[Mesh]) OR “protein C-reactive”[Title/Abstract]) OR (“inflammation”[Mesh] OR “inflammation”[Title/Abstract])) AND ((“exercise”(Mesh] OR “exercise”[Title/Abstract]) OR (“training”(Mesh] OR “training”[Title/Abstract]) OR (“aerobic exercise”[Mesh] OR “aerobic exercise”[Title/Abstract]) OR (“aerobic training”[Mesh] OR “aerobic training”[Title/Abstract]) OR (“endurance exercise”[Mesh] OR “endurance exercise”[Title/Abstract]) OR (“endurance training”[Mesh] OR “endurance training”[Title/Abstract]) OR (“resistance exercise”[Mesh] OR “resistance exercise”[Title/Abstract]) OR (“resistance training”[Mesh] OR “resistance training”[Title/Abstract])) AND ((“Fat accumulation”(Mesh] OR “fat accumulation”[Title/Abstract]) OR (“Obesity”(Mesh] OR “obesity”[Title/Abstract]) OR (“Overweight”(Mesh] OR “overweight”[Title/Abstract])))

Supplementary Table S2. PEDro Scale for quality assessment of the studies included in the systematic review.

Study	1	2	3	4	5	6	7	8	9	10	11	Score
Ahmadizad et al. (2015)	Yes	0	0	1	0	0	0	1	1	1	1	5
Arikawa et al. (2010)	Yes	1	0	1	0	0	0	0	1	1	1	5
Auerbach et al. (2013)	Yes	1	NA	1	0	0	1	0	1	1	1	6
Besse-Patin et al. (2013)	Yes	NA	NA	NA	0	0	0	1	1	NA	1	3
Brunelli et al. (2015)	Yes	1	0	1	0	0	0	0	0	1	1	4
Bruun et al. (2005)	Yes	NA	NA	NA	0	0	0	1	1	NA	1	3
Christiansen et al. (2010)	Yes	1	0	0	0	0	0	0	1	1	1	4
Duzova et al. (2018)	Yes	0	0	0	0	0	0	1	1	1	1	4
Dvorakova-Lorenzova et al. (2005)	Yes	NA	NA	NA	0	0	0	1	1	NA	1	3
Esposito et al. (2003)	Yes	1	1	1	0	0	1	1	1	1	1	8
Gram et al. (2017)	Yes	1	0	1	0	0	0	0	0	1	1	4
Ho et al. (2013)	Yes	1	0	1	0	0	0	0	1	1	1	5
Jae et al. (2006)	Yes	0	0	1	0	0	0	1	1	1	1	5
Khoo et al. (2015)	Yes	1	1	1	0	0	1	1	1	1	1	8
Klimcakova et al (2006)	Yes	NA	NA	NA	0	0	0	1	NA	NA	1	2
Kolahdouzi (2019)	Yes	1	0	1	0	0	0	1	1	1	1	6
Kondo et al. (2006)	Yes	0	0	NA	0	0	0	1	1	1	1	4
Lakhdar et al. (2013)	Yes	1	0	0	0	0	0	1	1	1	1	5
Leggate et al. (2012)	Yes	NA	NA	NA	0	0	0	1	1	NA	1	3
Loria-Kohen et al. (2013)	Yes	1	0	1	0	0	0	0	1	1	1	5
Marcell et al. (2005)	Yes	1	0	1	0	0	0	1	1	1	1	6
Moghadasi et al. (2012)	Yes	1	0	0	0	0	0	1	1	1	1	5
Nikseresht et al. (2014)	Yes	1	0	0	0	0	0	1	1	1	1	5
Nikseresht et al. (2018)	Yes	1	0	1	0	0	0	1	1	1	1	6
Olson et al. (2007)	Yes	1	0	1	0	0	0	1	1	1	1	6
Polak et al. (2006)	Yes	NA	NA	NA	0	0	0	1	1	NA	1	3
Vella et al. (2017)	Yes	1	0	1	0	0	0	1	1	1	1	6

NA, not applicable.

Supplementary Table S3. Effects of training on the circulating concentrations of IL-1β in sedentary adults with overweight and obesity.

Study	Subjects	Experimental conditions	Training protocol	Pre- vs Post-training differences	
				Fat Mass (%)	IL-1β (pg/ml)
Klimcakova et al. 2006	Men (50.4±2.3 yr) (N=12)	EC1: RT (33.6±1.2 kg/m²; N=12)	12 weeks 3 days/week 60 min/session 17 exercises, 1x12-15 at 60-70% RM	EC1: 31.6±4.9 vs 30.1±4.2	EC1: 1.6±1.2 vs 1.0±0.5
Auerbach et al. 2013	Men (20 to 40 yr) (N=48)	EC1: ET (28.1±1.3 kg/m²; N=12) EC2: Control (28.1±1.3 kg/m²; N=12)	12 weeks 7 days/week 65-85% HRR (600 kcal)	EC1: 31.3±4.1 vs 29.4±3.8* EC2: 31.3±4.1 vs 31.1±3.5	EC1: 0.9±1.0 vs 0.7±0.2 EC2: 0.9±1.0 vs 0.8±0.9
Tartibian et al. 2015	Postmenopausal women (50 to 65 yr) (N=28)	EC1: ET (25.5±3.9 kg/m²; N=14) EC2: Control (25.0±3.1 kg/m²; N=14)	16 weeks 3-4 days/week 25-30 min/session Treadmill 55% HR_{max}	EC1: 27.4±4.7 vs NR EC2: 26.4±5.4 vs NR	EC1: 3.6±0.7 vs 2.1±0.9*# EC2: 3.5±0.6 vs 3.7±0.5

EC=experimental condition; ET=endurance training; HR_{max}= maximal heart rate; HRR=heart rate reserve; NR= non-reported; RM=maximal repetition; RT, resistance training; * P<0.05 within group comparison. # P<0.05 between groups comparison (vs control). Data are shown as mean ± SD.

Supplementary Table S4. Effects of training on the circulating concentrations of IL-1ra in sedentary adults with overweight and obesity.

Study	Subjects	Experimental conditions	Training protocol	Pre- vs Post-training differences	
				Fat Mass (%)	IL-1ra (pg/ml)
**Auerbach et al. 2013**	**Men (20 to 40 yr) (N=48)**	**EC1: ET (28.1±1.3 kg/m²; N=12)** **EC2: Control (28.1±1.3 kg/m²; N=12)**	12 weeks 7 days/week 65-85% HRR (600 kcal)	EC1: 31.3±4.1 vs 29.4±3.8* EC2: 31.3±4.1 vs 31.1±3.5	EC1: 251±120 vs 227±109 EC2: 251±120 vs 239±94

EC=experimental condition; ET=endurance training; HRR=heart rate reserve; NR= non-reported; * $P<0.05$ within group comparison. Data are shown as mean ± SD.

Supplementary Table S5. Effects of training on the circulating concentrations of IL-8 in sedentary adults with overweight and obesity.

Reference	Participants	Experimental conditions	Training protocol	Pre- vs Post-training differences	
				Fat Mass (%)	IL-8 (pg/ml)
Bruun et al. 2005	Men (N=11) & Women (N=12) (NR yr)	EC1: ET (45.8±1.9 kg/m²; N=23)	15 weeks<br>5 days/week<br>2-3 h/session<br>Moderate intensity (NR)	EC1: 46.0±2.5 vs 41.1±2.3*	EC1: 5.6±0.6 vs 4.8±0.4*
Vella et al. 2017	Men (N=7) and Women (N=10) (18 to 44 yr)	EC1: HIIT (29.9±3.3 kg/m²; N=8)<br>EC2: ET (33.1±6.0 kg/m²; N=9)	8 weeks<br>4 days/week<br>30 min/session<br>EC1: 10 x (60s at 75-80% HRR + 60s at 35-40% HRR)<br>EC2: 55-59% HRR	EC1: 35.2±6.8 vs NR<br>EC2: 35.3±7.2 vs NR	EC1: 10.7±1.0 vs 8.6±1.0<br>EC2: 8.2±1.0 vs 11.7±0.9

EC=experimental condition; ET=Endurance training; HIIT=high-intensity interval training; HRR=heart rate reserve; NR=not reported; * P<0.05 within group comparison. Data are shown as mean ± SD.

Supplementary Table S6. Effects of training on the circulating concentrations of MCP-1 in sedentary adults with overweight and obesity.

Study	Subjects	Experimental conditions	Training protocol	Pre- vs Post-training differences	
				Fat Mass (%)	MCP-1 (pg/ml)
Bruun et al. 2005	Men (N=11) & Women (N=12) (NR yr)	EC1: ET (45.8±1.9 kg/m²; N=23)	15 weeks 5 days/week 2-3 h/session Moderate intensity (NR)	EC1: 46.0±2.5 vs 41.1±2.3*	EC1: 141±8.3 vs 122±6.3*
Christiansen et al. 2010	Men (N=40) and Women (N=34) (18 to 45 yr)	EC1: ET (33.3±4.0 kg/m²; N=25)	12 weeks 3 days/week 60-75 min/session (500-600 kcal)	NR	EC1: 244±86 vs 218±89*
Leggate et al. 2012	Men (23.7±5.2 yr) (N=12)	EC1: HIIT (29.1±3.1 kg/m²; N=12)	2 weeks 3 days/week HIIT 10 x (4 min 85% VO_{2peak} + NR R)	NR	EC1: 145±50 vs 128±38*
Auerbach et al. 2013	Men (20 to 40 yr) (N=48)	EC1: ET (28.1±1.3 kg/m²; N=12) EC2: Control (28.1±1.3 kg/m²; N=12)	12 weeks 7 days/week 65-85% HRR (600 kcal)	EC1: 31.3±4.1 vs 29.4±3.8* EC2: 31.3±4.1 vs 31.1±3.5	EC1: 102±36 vs 101.3±42.3 EC2: 102±36 vs 108.4±41.5

EC=experimental condition; ET=Endurance training; HIIT=high intensity interval training; R=rest between series; HRR=heart rate reserve; HR_{max}=maximal heart rate; VO_{2peak}=peak oxygen uptake; NR=non-reported; ~=estimated data; * P<0.05 within group comparison. Data are shown as mean ± SD.
